# The Effect of Vitamin D Supplementation for Bone Healing in Fracture Patients: A Systematic Review

**DOI:** 10.1155/2023/6236045

**Published:** 2023-02-28

**Authors:** Thomas Gatt, Adriana Grech, Homa Arshad

**Affiliations:** ^1^Mater Dei Hospital, Msida, Malta; ^2^Queen Mary University, London, UK

## Abstract

While most literature on vitamin D supplementation in fracture patients focuses on fracture prevention, the effect of vitamin D on bone healing is a much less studied concept. The primary aim of this systematic review was to assess whether vitamin D supplementation in fracture patients improves clinical or radiological union complications. The secondary aims were to assess supplementation effect on patient functional outcome scores and bone mineral density (BMD). A systematic search of all relevant articles was performed using the following databases: MEDLINE, Embase, Google Scholar, and Web of Science. The population selection included human patients with a fresh fracture treated conservatively or operatively. The intervention included any form of vitamin D supplementation, compared to no supplementation or a placebo. The primary outcomes assessed were clinical or radiological union rates or complications arising from the nonunion. The secondary outcomes assessed were functional outcome scores, BMD scores after treatment, and pain scores. A total of fourteen studies, assessing a total of 2734 patients, were included. Eight studies assessed the effect of vitamin D on clinical or radiological union. Five studies reported no significant difference in complication rates when supplementing fracture patients. Alternatively, three studies reported a positive effect with supplementation between the groups. One of these studies found a difference only for early orthopaedic complications (<30 days), but no differences in late complications. The other two studies found significant differences in clinical union; however, no changes were observed in radiological union. Six studies investigated functional outcome scores after supplementation. Four of these studies found no significant differences between most functional outcome scores. Only three studies reported BMD outcomes, one of which found limited effect on total hip BMD. The overall findings are that vitamin D alone does little to influence fracture healing and subsequent union rates or functional outcome. The studies suggestive of a positive effect were generally of a lower quality. Further high quality RCTs are needed to justify routine supplementation at the time of fracture.

## 1. Introduction

The role of vitamin D in calcium homeostasis and subsequent bone mineralisation is well established [1]. Despite this, the prevalence of vitamin D deficiency has reached epidemic proportions [2]. Over the past decade, a combination of increased awareness and testing has brought vitamin D deficiency to the forefront, leading to a significant increase in the amount of literature available [3]. Much of the literature in relation to fractures focuses on the effect of vitamin D in preventing risk. Two landmark meta-analyses by Bischoff-Ferrari et al. have showed that vitamin D supplementation appears to reduce fracture risk in the elderly [4, 5]. These recommendations have formed the basis of multiple guidelines on routine vitamin D supplementation despite being contested by some authors [6].

However, the effect of vitamin D on bone healing following a fracture is a much less studied concept. Studies performed in animal models have shown promising effects that adequate supplementation could enhance bone healing [7, 8]. From a biochemical aspect, vitamin D appears to be involved in every phase of the fracture healing process by mobilizing calcium. However, there is conflicting data showing varying levels of metabolites during the healing stage and the mechanism is poorly understood. The systematic review by Gorter et al. in 2014 was one of the first to broadly examine the effect of vitamin D on bone healing [9]. At the time, despite a total of 105 studies, no clinical studies focusing solely on vitamin supplementation were found. Only three human studies assessed the effect of vitamin D on bone healing, two of which had calcium cosupplementation and one showed an increase in the callus area at the fracture site [10–12]. Sprague et al. conducted a similar review in 2017, where they found that there is a high prevalence of hypovitaminosis D in a fracture cohort, however only identified a conference abstract which found a positive effect on bone healing [13]. Since then, this abstract has been published in its entirety by Haines et al. who concluded no difference with vitamin D supplementation [14]. Calcium and vitamin D supplements are highly cost-effective and could save almost up to €6 billion annually in the EU on fracture prevention [15]. Fracture complications are associated with high patient morbidity and increased hospital expenses [16]. If vitamin D was to show a positive effect on reducing fracture complications, this would be a highly cost-effective option to decrease both morbidity and healthcare resource expenditures [17].

With the increase in published research since the work by Gorter and Sprague, a repeated systematic review to assess more current literature would shed more light on this topic. In this review, importance was placed solely on the effect of vitamin D, without the confounding effect of combined calcium supplementation. The primary aim of this systematic review was to assess whether vitamin D supplementation in fracture patients improves clinical or radiological union complications. The secondary aims were to assess supplementation effect on patient functional outcome scores and bone mineral density (BMD).

## 2. Methods

The systematic review was performed in accordance with the Preferred Reporting Items for Systematic Reviews and Meta-Analysis (PRISMA) guidelines [18]. The review was registered on the PROSPERO database (CRD: 42022306990). A systematic search of all relevant articles was performed by using the following databases: MEDLINE, Embase, Google Scholar, and Web of Science from incepeption till 28th February 2022. The search strategy, included keywords, search terms, and MeSH headings, found in Appendix. Boolean operators AND/OR were used to expand and refine the searches where the databases allowed. Conference abstracts published by the Orthopaedic Research Society, the Orthopaedic Trauma Association, the European Federation of National Associations of Orthopaedics and Traumatology (EFORT), and the Orthopaedic Proceedings Supplement issued by The Bone & Joint Journal were also searched to include gray literature. The latter contains abstracts of articles presented at scientific meetings or congresses organised by various orthopaedic associations. A manual citation search of all included studies was performed.

### 2.1. Study Eligibility Criteria

The PICOS model was used to formulate the eligibility criteria for the included studies [19]. The population selection included human patients with a fresh fracture treated conservatively or operatively. The intervention included oral, liquid, or intramuscular form of vitamin D supplementation, compared to no supplementation or a placebo, regardless of dosing regimen. Studies where vitamin D was cosupplemented were only included if the calcium regimen was standard across all intervention groups including the control, or if the effect of vitamin D alone could be compared to control groups with no treatment. Observational studies with no intervention, which correlated vitamin D levels on admission and fracture outcomes, were excluded. The primary outcomes assessed were clinical and radiological union rates, or complications arising from the subsequent nonunion. Secondary outcomes assessed were functional outcome scores, BMD scores after treatment, and pain scores. All study types were included except for case series or case reports. There were no date or language restrictions. Studies involving animals, maxillofacial fractures, and pathological fractures or those involving fracture healing following elective primary surgery (e.g., osteotomy or ankle fusion) were excluded.

### 2.2. Study Selection and Data Collection

**Figure 1:**
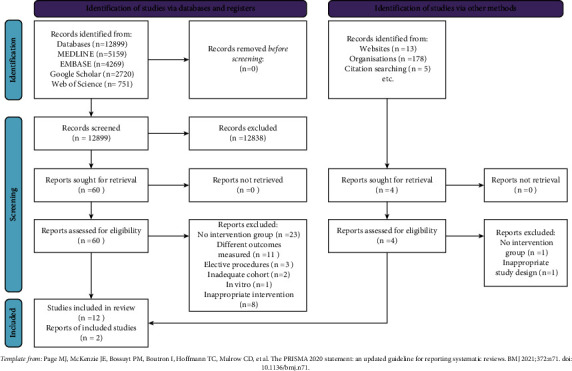
PRISMA flowchart summarizing screening process.

The study selection was first screened by title and then by abstract. The screening was performed by two authors (T.G and A.G), with a third author (H.A) to settle any disagreements following a discussion; however, this was not required. Cohen's kappa coefficient was 0.82 for title screening and 0.86 for abstract screening. The selection process is summarized in the PRISMA flow diagram (Figure 1). A total of 12,899 titles were identified throughout the screening process, of which 60 abstracts were identified. Of the abstracts excluded during this phase, 23 were studies assessing the effects of vitamin D status at the time of fracture on healing outcomes, with no intervention given. A total of eleven studies focused on alternative outcomes such as mortality, vitamin D metabolite levels, or bone turnover markers. Another eight studies had an inappropriate intervention which included vitamin D combined with calcium, which could not be compared separately. Three studies focused on outcomes after elective surgery such as ankle fusion or elective osteotomy, which were also excluded.

Data was extracted by the primary author (T.G) using the data template developed by the Cochrane Consumers and Communication Review Group. Attempts to obtain any missing data were made by contacting the corresponding author of the respective study through email. Any duplicate studies in the selection process were removed manually during abstract screening.

### 2.3. Quality Assessment

Risk of bias assessments was performed in duplicate using the Risk of Bias Version 2 (ROB-2) [20] tool for randomised control trials, the Risk of Bias in Nonrandomised Studies of Interventions (ROBIN-I) [21] tool for nonrandomised control trials, and the Newcastle-Ottawa Scale (NOS) [22] for observational studies by two authors (T.G & A.G). For RCTs, the quality assessments included bias assessments for intervention deviation, missing outcomes, outcome measurements, and result selection. For NRCTs, bias assessment for confounding, intervention classification, and patient selection was also performed in addition to the assessments for RCTs. For cohort studies, the quality assessment domains included cohort selection, cohort comparability, and outcome reporting.

### 2.4. Data Synthesis

The patient and study characteristics were reviewed by the two authors (T.G and A.G). There was a high degree of heterogeneity noted amongst the studies included. When assessing union rates, the studies focused on a variety of age groups, with different fracture types, different supplementation regimens, and different time endpoints for the primary outcome. The secondary outcomes assessing functional scores were also very heterogenous, with only two studies reporting the same functional outcome. As a result of this, a meta-analysis was not possible and a descriptive review of the collated data was instead presented throughout the study.

## 3. Results

A final total of 14 studies, assessing a total of 2734 patients, were included to assess fracture outcomes following vitamin D supplementation [10, 14, 23–34]. Nine of these were controlled trials of which seven were randomised and two were nonrandomised. Five studies were cohort trials of which one was prospective and four were retrospective. One study identified in abstract form during the search was later published in full and also included in the analysis [34].  Table 1 summarizes the characteristics of the included studies, in order of the outcome investigated, and outlines the main findings.

### 3.1. Supplementation Strategy

There was a wide variation in vitamin D supplementation techniques amongst the studies. The research identified bolus dosing regimens ranging from 100,000 to 300,000 IU [14, 27, 28], daily dosing regimens ranging from 800 to 2000 IU daily [23–25, 29–31], or a combination of bolus and daily dosing [26, 33, 34]. Supplementation forms included oral tablet, liquid drops, or intramuscular injections. Vitamin D3 was used for supplementation in all studies except for Hiokka et al. who used alfacalcidol [10].

### 3.2. Fracture Union

**Figure 2:**
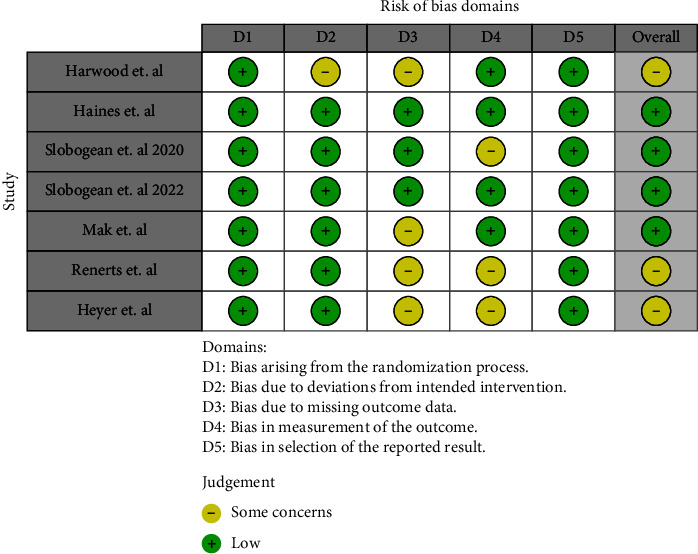
ROB-2 analysis for randomised control trials.

**Figure 3:**
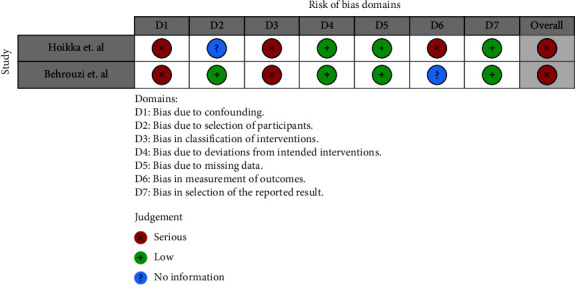
ROBIN-I analysis for nonrandomised control trials.

Eight studies assessed the effect of vitamin D on clinical or radiological union [14, 24, 27, 31–34]. There were two studies which did not distinguish between the union types [28, 32]. Of the remaining studies, four reported both radiological and clinical outcomes [14, 31, 33, 34], while two studies reported only radiological outcomes [24, 27]. There were five studies which reported no significant difference in complication rates when supplementing fracture patients [14, 24, 27, 32, 34]. The overall quality of these studies was rated as “low risk of bias” for the controlled trials and “fair quality” for the observational studies (Figures 2 and 3 and Table 2). There were three studies which reported a positive effect with supplementation between the groups [28, 31, 33]. One study found a difference only in early orthopaedic complications (<30 days) but did not find any differences in late complications [28]. Another two studies found statistically significant differences in clinical union, however no changes in radiological union [31, 33]. The quality of these studies ranged from “poor” to “fair” for the two observational trials, and a “serious risk of bias” was observed in the nonrandomised control trial (Figures 2 and 3 and Table 2).

### 3.3. Functional Outcome

A total of six studies reported a wide variety of patient functional outcome scores, reported at varying stages of the recovery period [10, 23, 25–27, 29]. These scores included the 12-Item Short Form Physical Component scores (SF-12-PC), Owestry Disability Index (ODI), Roland Morris Disability Questionnaire (RMDQ), 36-Item Short Form Scores (SF-36), EuroQol 5D-3L score, gait velocity, grip strength, Barthel Index (BI), and Patient-Rated Wrist Evaluation (PRWE) scores. Only two studies found significant differences in the functional outcome scores they assessed. One study found supplementation improved SF-12-PC scores at one year (*p*=0.003) [23]. Another study found that although no differences in the initial rehabilitation phase were found, supplementation slowed the decline in EQ (5D) scores after six months [25]. The remaining four studies found no significant differences between the majority of functional outcome scores [10, 26, 27, 29]. The study by Ko et al. was graded as “fair,” while the control trials ranged from “low risk of bias” to “serious concerns” [27]. The latter refers to Hoikka et al.'s study which assessed the grip strength [10]. One study found no improvement on gait velocity (*p*=0.490), but a decrease in pain scores at 28 weeks when supplementation was used (*p*=0.037).

### 3.4. Bone Mineral Density

Only three studies reported BMD outcomes. Harwood et al. found that vitamin D had a small but statistically significant effect on total hip BMD at 28 days, but not in spine BMD in hip fracture patients [30]. Heyer et al. found no difference in BMD between high or low dose supplementation versus a control for conservatively managed distal radius fractures measured at two, four, six, and eight weeks postfracture [29]. Harwood et al. found no changes in the nondominant distal radius of hip fracture patients at three or six months between groups [30].

## 4. Discussion

The overall impression of these findings is that vitamin D does very little to influence fracture healing and subsequent union rates. Two of the three studies which concluded a positive effect of supplementation were generally of a lower quality [31, 33]. The nonrandomised study by Behrouzi et al. saw the authors dividing the groups based on an undocumented vitamin D level and only supplemented the deficient patients with a bolus dose. In a retrospective study by Gorter et al., the groups were misbalanced with nonsupplemented patients (*n* = 368) outnumbering the supplemented patients (*n* = 141). The main differences in this study are seen only in the smaller cohort of thirty patients which remained deficient despite treatment. These patients may either have required higher supplementation in view of severe deficiency or were not fully compliant to the treatment.

The phase II pilot RCT by Slobogean et al. in 2022 serves as a high quality benchmark for future work on the topic [34]. The authors investigated four groups to assess not only effect of vitamin D on healing but also the effect of its dose, which was a persistent problem amongst the other studies due to a wide variation. This study is also unique in which the authors have utilised validated scoring systems to assess clinical (FiX-IT) [35] and radiological union (RUST) [36]. This may aid in reducing observation bias for what may be a challenging outcome to assess, as well as provide a numerical outcome which may be pooled in future studies on the topic. Slobogean and colleagues found no improvement on radiological or clinical difference in fracture healing at 3 or 12 months when comparing high dose with low dose, high loading dose with high daily dose, and low dose with placebo. The only significant difference found was in post-hoc analysis for clinical union scores at 3 months when comparing high dose to placebo (*p* = 0.16), however required confirmation with a larger trial, especially in keeping with the rest of the negative findings.

When assessing the primary outcomes of clinical and radiological union, the decision was made to include two studies which assessed our primary outcome in combination with other orthopaedic complications such as peri-implant fractures, dislocations, wound infections, and reoperations [24, 28]. While these outcomes may be influenced by factors other than bone healing, such studies were included to not lose quality evidence on the topic. The justification for inclusion was that incidence of these additional complications was low, except for wound infection which may be identified by the timing of complication manifestation. Regardless of this, results from these studies should be interpreted with caution. For instance, the Ingstad et al. study reports a difference in early complications (<30 days) of borderline statistical significance (*p*=0.044). Early complications would favour surgical skin infections as opposed to the bone healing complications which is the scope of this review. Furthermore the late complications at 12 weeks (*p*=0.242) and 1 year (*p*=0.079) were not found to be significant and would typically include union complications.

The included studies in this review had a large degree of heterogeneity in both patient characteristics and study design. Different cohorts of patients were assessed, ranging from young trauma patients with tibial or femur shaft fractures to middle aged women with distal radii fractures and to elderly patients with neck of femur fractures. This heterogeneity was also seen in supplementation strategy. Numerous organisations have issued varying recommendations on the optimal advised intake, as well as cutoffs for vitamin deficiency [37–39]. These vary based on their geographical prevalence, as well as whether the guidelines focus on bone or pleiotropic effects. There is no standard recommended daily intake dosing, and the advice is that supplementation should be patient-specific and preferably adhering to regional guidelines [40]. Some of our included studies made use of bolus dosing which may improve compliance and achieve greater production of vitamin D3 faster, when compared to daily dosing. The latter, however, offers more predictable and long-lasting effects [41]. There thus appears to be no widely accepted supplementation strategy yet, and no positive correlation with a particular regimen was identified in this review.

With respect to secondary outcomes, most functional scores showed no benefit with supplementation. There was a wide amount of variety in the scoring systems reported, with six studies looking at eleven functional outcome scores. One of these studies, although finding no significant difference in their outcome scores, reported an improvement in the pain component of EuroQoL scoring system at 26 weeks [26]. This was not replicated in the rest of the scoring systems, the majority of which included pain components. The rationale of a presumed positive impact of vitamin D on functional outcome may be due to its effects on muscle strength performance rather than bone healing. This correlation has been studied extensively in the literature on both athletes and frail elderly patients to assess fall risk reduction [42–44]. A review of this literature shows a number of studies which support this positive correlation; however, difficulties with data aggregation and a number of studies conversely not corroborating these findings mean that the data here are still conflicting [45, 46]. For instance, in a study by Lee et al., vitamin D levels correlated with improved grip strength in the unaffected hand of distal radius fracture patients [47]. Yet in our included studies, active supplementation in neck of femur fractures found no significant improvement in grip strength at one, three, or six months [10, 26] nor in gait velocity [26].

Hypovitaminosis D appears to be highly prevalent in fracture patients [48]. A study by Gorter et al. demonstrated that vitamin D status at the time of fracture may impact fracture healing [49]. This study only found a difference in clinical union, with no difference in radiological union. Our review excluded several recent observational studies assessing vitamin D status on outcomes, which were beyond the scope of our aims as they lacked an intervention. A more recent systematic review of this literature could be the scope of further research. It may well be that although commencing supplementation at the time of a fracture is too late, supplementing to normal vitamin D levels before would improve outcomes if a fracture was to occur.

### 4.1. Strengths and Limitations

To our knowledge, this systematic review is the first since the two major systematic reviews on this topic by Gorter et al. and Sprague et al. in 2014 and 2017, respectively. Our systematic review is strengthened by its comprehensive search through multiple databases and gray literature sources. It also includes most studies published after 2017, which had not been previously considered. It also excludes studies using cosupplementation of calcium, such as the Doetsch et al. study which had been included in other reviews [11]. The included studies capture a broad international cohort of patients, of varying ages and fracture types, which was a fair representation of fracture patients.

The main limitation of our study is the wide degree of heterogeneity in many of the study designs, including treatment supplementation strategies and outcomes measured. Different studies assessed union or functional outcomes at different time points or by different criteria. Only one study employed a numeric scoring system for union assessment [34]. As a result, the data for union rates between treatment and nontreatment groups were unable to be pooled, permitting only the assessment of trends. Several functional outcome scores were self-reported, as was compliance to treatment in certain studies. Both these factors may lead to response bias. Another limitation is the inclusion of nonrandomised control trials or retrospective studies. This evidence is generally of a lower quality and could again potentially increase the risk of bias. Had this systematic review been solely limited to RCTs, none would have shown any positive effect of supplementation on union or functional outcomes. A further limitation of this review is that the studies are underpowered. Incidence of fracture healing complications is generally low, so large numbers of patients would be required, incurring significant costs and limiting feasibility. Indeed, this was the case for several studies which halted recruitment early or did not proceed beyond the pilot trial. In this case, the risk of type 2 errors is increased and could alter results.

## 5. Conclusion

In summary, it appears that despite initial successes in animal studies, the supplementation of vitamin D in an effort to promote fracture healing and subsequent outcomes does not translate to humans in clinical practice. There has been significantly more literature on the topic in recent years although the evidence is still plagued by underpowered studies. The standards for any future quality research on the topic should include power calculations and a feasibility study taking into consideration the regional research framework. This should then be followed by a multicentre, randomised control trial of fracture patients, supplemented with varying doses of vitamin D supplementation, and independently assessing union at regular intervals using validated scoring systems.

While hypovitaminosis D is prevalent in fracture patients, the administration of vitamin D alone at the time of diagnosis does not confirm any additional benefit. Of the studies which do show some positive effect for supplementation, these are undermined by nonrandomisation and retrospectivity. Reviewing the literature to determine the effect of vitamin D status at fracture diagnosis is warranted given the increase of recent published studies. Until more high quality and adequately powered trials emerge with evidence to the contrary, routine supplementation of vitamin D alone at the time of fracture diagnosis appears futile in improving bone healing.

## Figures and Tables

**Table 1:** Study design characteristics and main findings.

Author	Country	Study design	Patient cohort	Cohort size	Intervention	Outcomes assessed	Summary of results
Haines et al. [14]	USA	Double blind RCT	18 yr+; tibia, humerus, femur diaphyseal fractures	100	Deficient patients given either 100,000 IU oral D3 or placebo	Clinical and radiological union	No difference between union rates between treatment and control (*p*=1.000)

Slobogean et al. [24]	USA/Canada	Double blinded pilot trial for FAITH2	18–60 yr; NOF part of the FAITH2 trial; randomized to either CS or SHS	86	D3 drops 2000 IU BD for 6 months vs. placebo	Orthopaedic complications including reoperation, head osteonecrosis, malunion, and radiological nonunion	Rate of nonunion was 8.7% (*n* = 4) vs. 7.5% (*n* = 3) when comparing supplementation to placebo, respectively
							For overall complications, the hazard ratio was 0.96 for supplementation (95% CI 0.42–2.18) (*p*=0.92) and this study was underpowered

Slobogean et al. [34]	USA	Double blind RCT (vita shock trial)	18–55 yr; tibia or femoral shaft fracture for intramedullary nail	102	Three month supplementation across four treatment groups; 150,000 IU loading at injury and 6 weeks vs. 4000 IU daily vs. 600 IU daily vs. placebo	Clinical and radiological union (FIX-IT and RUST scores)	No differences between clinical or radiological union between loading doses vs. high daily dosing; high dose vs. low dose groups or between low dose vs. placebo at 3 or 12 months
							Post-hoc comparison of high dose vs. placebo showed improved clinical union (*p*=0.16) but not radiological union (*p*=0.76)

Behrouzi et al. [33]	Iran	Nonrandomised, double blind trial	60 yr+; intertrochanteric fractures	100	Participants were divided into two groups based on vitamin D status. All patients given 50000 D3 IM bolus, but deficient patients were supplemented with 50,000 IU oral weekly for 12 weeks	Clinical and radiological union	No significant difference in radiological union rate at 2, 4, 8, or 12 weeks (*p* > 0.05)
							There was significant difference in clinical union at 4 (*p*=0.005) and 8 weeks (*p*=0.036) favouring vitamin D supplementation

Ko et al. [27]	Korea	Prospective cohort	Osteoporotic vertebral fractures	130	Groups weredivided into supplemented (*n* = 65) and nonsupplemented (*n* = 65). Supplementation was 300,000 IU D3 SC for deficient or 100,000 IU for insufficient	Radiological union, functional outcome scores (ODI, RMDQ), and QoL scores (SF-36)	Fracture union in all patients regardless of vitamin D level. No significant difference in functional outcomes (ODI, *p*=0.144; RMDQ, *p*=0.194 or QoL scores (SF-36 PC,), *p*=0.934) between the supplemented and nonsupplemented group
Gorter et al. [31]	Netherlands	Retrospective cohort	18 yr+; upper or lower extremity fracture	617	Deficient patients were supplemented with 1200 IU oral D3 daily	Clinical and radiological union	Patients remaining vitamin D deficient despite supplementation had a higher rate of delayed clinical union (*p* < 0.001) but not radiological union. (*p*=0.67)

Ingstad et al. [28]	Norway	Retrospective cohort	18 yr+; hip fractures for operation	407	100,000 IU oral D3 loading dose	Orthopaedic complications (incl nonunion, SSI < dislocation and peri-implant fractures)	Decrease in early (<30 days) orthopaedic complications only, with loading dose. (*p* − 0.044)

Bodendorfer et al. [32]	USA	Retrospective cohort	18 yr+; fracture type unknown	201	1000 IU D3 and 1500 mg Ca for all patients. Deficient or insufficient patients were given 50000 D2 weekly until normal D levels or healing demonstrated	Healing complications; nonunion, malunion, delayed union, wound problems, or infection	No significant difference between initial (*p*=0.92) or repeat (*p*=0.91) vitamin D in patients who developed fracture complications

Mak et al. [26]	Australia	Double blind RCT	65 yr+; NOF for surgery	218	All patients had oral 800 IU and 500 mg. Supplementation groups had oral loading 250,000 IU D3 vs. placebo	Gait velocity, grip strength, and BI, (EguroQol EQ5D)	No significant differences in gait velocity between both groups. No significant differences for BI (*p*=0.96) and grip strength (*p*=0.815) at 4 weeks between the groups
							EuroQoL scores were higher in treatment group but not significant. (*p* 0.092) pain scores at week 26 were significant higher in treatment group (*p*=0.037)

Renerts et al. [25]	Switzerland	Double blind RCT	65 yr+; NOF surgery	173	Patients had baseline 800 IU D3 and 1 g Ca. Then, supplementation group was given D3 2000 IU/dly + -HE vs. no further supplementation + -HE	Health-related quality of life (EuroQol-EQ5D)	No difference in quality of life between two interventions over time (*p*=0.9); however, high dose vitamin D slowed health-related quality of life decline after 6 months (*p*=0.03)

Heyer et al. [29]	Netherlands	Single blind RCT	50 yr+; females with conservative distal radius fractures	32	Three groups given either high dose (1800 IU daily), low dose (700 IU daily), and no treatment. Liquid vitamin D was administered at 2 boluses on weeks 1 and 6	BMD using HRpQCT; PRWE scores	No difference in total BMD between control vs. low dose (*p* 0.26) or high dose vs. control (*p*=0.388)
							No difference in PRWE scores between control vs. low dose (*p* 0.405) or high dose vs. control (*p*=0.249)

Sprague et al. [23]	Multiple	Retrospective cohort	50 yr+; NOF fractures (FAITH trial cohort)	573	1000 U daily to all patients then divided the groups by compliance (consistent, inconsistent, and no vit D use)	Short Form-12 Physical Component Score	Consistent vitamin D supplementation after fracture improved 1 year SF-12 Physical Component Scores. (*p*=0.003)

Harwood et al. [30]	UK	Nonblinded RCT	60 yr+; NOF admitted to orthogeriatric rehab ward	97	Four groups; 300,000 IU D2 injected vs. 300,000 IU D2 injected + 1 g/day Ca vs. 800 u/day D3 and 1 g/day Ca vs. no treatment	28 day BMD	Vitamin D had a small but statistically significant effect on total hip BMD at 28 days. Mean difference of 0.013 (95% CI interval; 0.003; 0.023). Mean difference increased when calcium was added. No differences for spine BMD

Hoikka et al. [10]	Finland	NRCT	50 yr+; NOF fracture	37	1 mcg daily alfacalcidol and 2.5 g caco_3_ vs. placebo and 2.5 g caco_3_	BMD and grip strength	No change in BMD or grip strength after 3 or 6 months

BI, barthel index; BD, bis in die; BMD, bone mineral density; CS, cannualted screws; HE, home exercise therapy; FIX-IT, function index for trauma; IM, intramuscular; HRpQCT, high resolution peripheral computed topography; NRT, nonrandomised control trial; NOF, neck of femur; ODI, Owestry Disability Index; PRWE, Patient-Rated Wrist Evaluation; RCT, randomised control trial; RMDQ, Roland Morris Disability Questionnaire; RUST, radiograph union scale in tibial fractures; SHS, sliding hip screws; SC, subcutaneous; SF, short form.

**Table 2:** Newcastle-Ottawa scale summary of observational studies.

Study	Selection	Comparability	Outcome	Overall
Ko et al.	★★★★		★★	6
Gorter et al.	★★★		★	4
Ingstad et al.	★★	★★	★	5
Bodendorfer et al.	★★★★		★	5
Sprague et al.	★★	★★	★	5

Overall score: poor < 4; fair 5-6; good > 7.

## Data Availability

The data used to support the findings of the study are obtained from the corresponding author upon request.

## Appendix

- MEDLINE:
- (fracture) AND ((adult) OR (PAED^*∗*^ OR (CHILD^*∗*^) OR (HUMAN) OR (“Bone and Bones”[Mesh])) AND ((“Vitamin D”[Mesh]) OR (CHOLECALCIFEROL) OR (ERGOCALCIFEROL) OR (CALCITRIOL) OR (1,25(OH)2D) OR (1,25-DIHYDROXYCHOLECALCIFEROL) OR (SUPPLEMENT^*∗*^)) AND ((HEAL^*∗*^) OR ^*∗*^(UNION) OR (NON-UNION) OR (DELAYED UNION) OR (MAL-UNION) OR (PAIN^*∗*^) OR (OUTCOMES) OR (CHRONIC PAIN) OR (“Fracture Healing”[Mesh])).
- EMBASE:
- “fracture”/exp AND “adult”/exp OR “child”/exp OR “human”/exp) AND (“vitamin d”/exp OR “colecalciferol”/exp OR “ergocalciferol”/exp OR “calcitriol”/exp) AND (“fracture healing”/exp OR “bone defect healing” OR “bone fracture healing” OR “bone healing” OR “bone healing, fracture” OR “bone union” OR “consolidation, fracture” OR “fracture bone healing” OR “fracture consolidation” OR “fracture healing” OR “fracture union” OR “healing, fracture” OR “fracture nonunion”/exp OR “delayed fracture healing” OR “delayed union” OR “fracture healing impairment” OR “fracture malunion” OR “fracture nonunion” OR “fracture union delay” OR “fractures, malunited” OR “fractures, ununited” OR “malunion” OR “malunited fractures” OR “non-union” OR “nonunion” OR “nonunion (fracture)” OR “nonunion fracture” OR “ununited fracture” OR “ununited fractures” OR “pain”/exp) AND [embase]/lim NOT ([embase]/lim AND [medline]/lim).
- Web of Science:
- Vitamin D AND healing.
- Google Scholar:
- (fracture) AND (adult OR PAED^*∗*^ OR CHILD^*∗*^OR HUMAN) AND (Vitamin D OR CHOLECALCIFEROL OR ERGOCALCIFEROL OR CALCITRIOL OR 1,25OH2D OR 1,25-DIHYDROXYCHOLECALCIFEROL OR SUPPLEMENT^*∗*^) AND (HEAL^*∗*^ OR UNION OR NON-UNION OR DELAYED UNION OR MAL-UNION OR PAIN^*∗*^ OR OUTCOME OR CHRONIC PAIN OR Fracture Healing).
